# The Impact of Resilience and Leader–Member Exchange on Actual Turnover: A Prospective Study of Nurses in Acute Hospitals

**DOI:** 10.3390/healthcare13020111

**Published:** 2025-01-08

**Authors:** Saori Yamaguchi, Yasuko Ogata, Miki Sasaki, Ayano Fujiyoshi-Ito, Yuki Yonekura

**Affiliations:** 1 Graduate School of Health Care Sciences, Institute of Science Tokyo, 1-5-45 Yushima, Bunkyo-ku, Tokyo 113-8510, Japan; yogata-tky@umin.ac.jp (Y.O.); mksasaki.tmdu@gmail.com (M.S.); ns190001@tmd.ac.jp (A.F.-I.); 2 Graduate School of Nursing Science, St. Luke’s International University, 10-1 Akashi-cho, Chuo-ku, Tokyo 104-0044, Japan; yyonekura@slcn.ac.jp

**Keywords:** turnover, nurse shortage, resilience, leadership, leader–member exchange

## Abstract

Background/Objectives: High nurse turnover has economic implications for healthcare organizations and impacts the quality of care. Individual, job-related, and organizational factors determine nurse turnover. This study, thus, aimed to investigate the impact of nurses’ resilience and the quality of the relationship between staff nurses and nurse managers, defined as leader–member exchange (LMX), on actual nurse turnover. Methods: A prospective study was conducted from June to July 2022, targeting nurses from three advanced treatment hospitals in Japan. Data were collected through a self-administered questionnaire investigating the participants’ actual turnover in April 2023. The data of 1130 nurses were analyzed in this study. A binary logistic regression analysis was performed using actual turnover as the dependent variable and nurse resilience and LMX as independent variables. Results: The results revealed that while LMX influenced actual turnover, nurse resilience had no statistically significant relationship with turnover. Conclusions: The findings show that LMX needs to be improved if actual turnover is to be curbed.

## 1. Introduction

The global shortage of nurses has become critical, with a projected deficit of 5.7 million nurses by 2030 [1]. Nurse turnover presents a significant challenge in human resource management. High nurse turnover rates lead to a reduction in the nursing workforce and inadequate staffing, imposing an increased workload on the remaining nurses and generating significant economic costs [2]. A diminished nursing workforce contributes to increased dissatisfaction, stress, and the development of burnout syndrome, among other issues. These factors negatively affect patient safety and compromise the quality of care [3]. To address the shortage of nurses, the World Health Organization estimates that the number of nursing school graduates will need to increase by an average of 8% annually by 2030 [1]. However, considering factors such as turnover, withdrawal from the nursing profession, and international relocation, relying solely on newly graduated nurses to meet workforce demands may be insufficient. Therefore, an effective strategy to address the shortage is to minimize turnover among nurses already working in healthcare and promote retention.

Various factors contribute to nurses’ intentions to leave employment, including individual, job-related, and organizational determinants [4]. Personal factors, such as job satisfaction, age, organizational commitment, resilience, and self-efficacy, play significant roles [5,6,7]. Interpersonal factors, including supervisors’ behaviors and leadership styles, also impact these intentions [4,8].

The COVID-19 pandemic has profoundly impacted nurses, changing the national and global economy, healthcare systems, and people’s lives. Nurses are frequently exposed to various work-related stress factors, including the demands of patient care, time-related pressures, and management responsibilities, which are further exacerbated by risks such as COVID-19 infection [9]. A recent study reported that nurses’ resilience is related to turnover intentions [10]. Resilience helps nurses cope and adjust positively, even under difficult circumstances, preventing them from leaving the profession [11]. Moreover, interventions can strengthen nurse resilience [12]. It is important to examine whether resilience, an individual factor that can intervene and may be associated with turnover, effectively reduced turnover during the COVID-19 pandemic.

Leadership, an organizational factor influencing employees’ intention to leave the organization, must be considered when reducing turnover [13]. While many leadership theories describe leadership as focusing on the leader’s personal characteristics and the characteristics of the situation, leader–member exchange (LMX) theory focuses on the quality of the dyadic relationship between leaders and followers [14]. One study has shown that high-quality leader–follower relationships exert a positive influence on the achievements of an organization and are inversely related to a nurse’s turnover intention [15]. LMX indicates the quality of relationships between staff nurses, who are followers, and nurse managers, who are leaders. Staff nurses with high LMX tend to receive abundant organizational information from nurse managers and build trust with them [16]. In situations where face-to-face contact with others must be avoided to prevent the risk of COVID-19 infection, staff nurses who receive more individualized support from the nurse manager—one of their few contacts on the job—and who perceive support from the organization may be more willing to stay on the job. Turnover results from an individual nurse’s choice; LMX focuses on the one-on-one relationship between the staff nurse and the nurse manager, so in situations with limited relationships with others, such as during the COVID-19 pandemic, LMX may influence nursing staff turnover. Investigating whether LMX effectively reduces nurse turnover in such situations is important.

Prior research has shown that nurses’ turnover intentions increase in stressful situations such as the COVID-19 pandemic [17]. Previous studies of nurse turnover have identified factors associated with turnover intention. However, turnover intention does not necessarily lead to actual turnover. Few studies have been conducted on actual turnover, and by investigating and clarifying the factors that contribute to actual turnover, it will be possible to consider specific measures to prevent turnover. Conducting research during situations such as the COVID-19 pandemic may lead to strategies to help organizations prepare for stressful situations in the future.

No studies have clarified a relationship between actual turnover and resilience or LMX during the COVID-19 pandemic; these associations need to be tested while the pandemic is still ongoing.

### Aim

**Figure 1:**
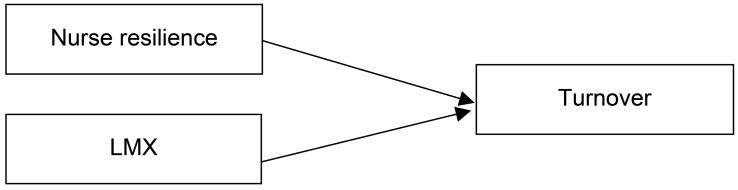
The research model. LMX: leader–member exchange.

This study aimed to identify whether nurse resilience and the quality of relationships between nurses and nurse managers (LMX) impact actual nurse turnover. To achieve this goal, we formulated the following hypotheses (Figure 1):

**Hypothesis** **1.** *Nurse resilience influences actual turnover*.

**Hypothesis** **2.** *The quality of the relationship between nurses and nurse managers (LMX) influences actual turnover*.

These hypotheses were formulated based on previous research findings on the influence of individual and organizational factors on nurse turnover [4] and the conservation of resources (COR) theory. The COR theory is a stress model related to motivation developed by Hobfoll (1989) [18]. It is based on the idea that humans strive to preserve and protect current resources and that the potential practical loss threatens the parties involved.

Nurses retain and protect resources, thus reducing turnover [19]. Resilience is psychological resources that enable individuals to bounce back and cope with negative situations [20]. Resilient nurses react to adverse situations by recognizing threats and investing the time and energy necessary to “bounce back” to equilibrium [21]. Resilience stops the loss of resources and helps to gain them [22]. Nurses with high LMX receive more interpersonal and organizational resources from their nurse manager. The nurse–nurse-manager relationship in LMX functions offers a supportive relationship in which resources are retained and replenished [23]. We hypothesized that resilience as a resource and LMX as a relationship that supports resource retention and replenishment would prevent nurse turnover during the COVID-19 pandemic.

In light of the above, Hypotheses 1 and 2 (Figure 1) were formulated for this study, referring to previous research on nurse turnover and COR theory and considering the context of the COVID-19 pandemic.

## 2. Materials and Methods

### 2.1. Study Design

This was a prospective survey using self-administered questionnaires. We requested that all 87 hospitals approved as advanced treatment hospitals in Japan participate in the survey. A letter asking hospitals to complete the survey was mailed to the target hospitals, and three agreed to participate. The survey was conducted using a questionnaire in two stages (Surveys 1 and 2). Advanced treatment hospitals are high-level acute care hospitals that provide advanced medical care and are included in the “List of Medical Institutions Approved as Advanced Treatment Hospitals (as of 1 November 2021)” published by the Ministry of Health, Labour and Welfare. In Japan, advanced treatment hospitals receive financial subsidies from the government to provide medical care to COVID-19 patients and are actively accepting COVID-19 patients. Differences between medical institutions in the medical care system, such as the workload in responding to COVID-19, patient background, infection control equipment, and number of medical professionals, may affect the reasons for nurses’ resignations. Because the survey was conducted during the COVID-19 pandemic, the target was advanced treatment hospitals where the medical care system is thought to be maintained at a certain level.

### 2.2. Participants and Data Collection

Survey 1 (conducted in June and July 2022) distributed a self-administered, unmarked questionnaire to all nurses and nurse managers in the three hospitals. The questionnaire measured demographic characteristics, resilience, and LMX. The target group included 2989 staff nurses, of whom 1405 completed the questionnaires (response rate: 47.0%). The target group also included 96 nurse managers, of whom 54 completed the questionnaires (response rate: 56.3%).

For Survey 2 (conducted in April 2023), the nursing management departments of the three cooperating hospitals provided IDs for all nurses who had resigned by 31 March 2023. Each hospital arbitrarily assigned these IDs to each subject, and participants entered their IDs in the Survey 1 questionnaire. Data combining the IDs answered in Survey 1, and the presence or absence of IDs answered in Survey 2 (with ID = resigned group, without ID = continuing group) were used in the analysis. Participants aged 64 years or older who had reached retirement age were excluded from the analysis; IDs were managed by the nursing administration department of the cooperating hospital so that researchers could not identify individuals by their IDs.

### 2.3. Measurements

#### 2.3.1. Demographic Characteristics

The questionnaire collected demographic characteristics, such as age, sex, number of years of nursing experience, and educational background. Previous studies have reported that these characteristics relate to actual turnover [24]. According to the labor force participation rate by age group, the rate of women’s regular employment peaks between the ages of 25 and 29 and declines with increasing age [25]. This reflects the fact that many women temporarily leave work, due to marriage and childbearing. Since approximately 91% of nurses in Japan are women, we can assume based on women’s labor force participation rate by age group that the number of nurses who quit working increases. The changes in the roles they play in society and personal living environments due to marriage may contribute to actual turnover. Therefore, in this study, we categorized the participants into the following age groups: 20–24, 25–29, 30–39, and over 40. The educational background of the survey participants was classified as “Associate degree or below” and “Bachelor’s degree or higher” based on several previous studies.

#### 2.3.2. Actual Turnover

In Survey 2, the nursing administration departments of the three hospitals provided ID numbers for participants who had resigned by March 2023 (survey date: April 2023). Participants whose ID numbers were provided by the hospital nursing departments were considered “resigned” and assigned a “1”. Participants whose ID numbers were not provided were assigned a “0” for “continuing” and used in the analysis.

#### 2.3.3. Nurse Resilience

Nurse resilience was measured using the Resilience Scale developed in Japan [26]. The scale used in this study was originally developed for adolescents, but it has also been used for people other than adolescents, such as teachers [27] and nurses [28]. With permission from the developer, we used it with the nurses who were the subjects of this survey. This scale consists of three subscales and 21 items: seven items measure novelty seeking, nine measure emotional regulation, and five measure positive future orientation. Responses were collected using a 5-point Likert scale, ranging from “5 = Yes” to “1 = No”. For reverse-scored items, the number of responses was reversed. The overall score was calculated by averaging the participants’ responses to the Resilience Scale. The maximum and minimum values were 5 and 1, respectively, with higher scores indicating greater resilience. The validity and reliability of the Resilience Scale were confirmed [26]. In this study, the Cronbach’s alpha for the entire 21-item scale was 0.894.

In selecting the resilience scales, we compared the meanings of items from several of the widely used resilience scales, such as the Bidimensional Resilience Scale (BRS) [29] and the Japanese version of the Resilience Scale (RS) [30], that we could collect from developers. Specifically, we examined whether the content was appropriate as an individual characteristic in the difficult situation of the COVID-19 pandemic. In other words, we examined whether the BRS, RS, and others collected were measures that capture the characteristics of positive coping and adaptation by nurses. Consequently, we selected a mental resilience scale with “novelty seeking”, “emotional regulation”, and “positive future orientation” as subfactors.

#### 2.3.4. LMX

The quality of the relationship between staff nurses and nurse managers was measured using the Multidimensional Measure of Leader-Member Exchange, Japanese version (LMX-MDM-J) [31], which is the Japanese edition of the LMX-MDM Measure [32]. The LMX-MDM-J is based on the concept that the quality of leader–member relationships is critical in how staff respond to the work environment [31]. This scale comprises 12 items across 4 subscales: professional respect, loyalty, affect, and contribution. Responses were obtained using a 7-point Likert scale, ranging from “7 = Strongly agree” to “1 = Strongly disagree”. The score across all items represented the LMX-MDM-J score. The maximum and minimum values were 7 and 1, respectively. The higher the score, the higher the relationship quality between nurses and nurse managers. The validity and reliability of the LMX-MDM-J have been confirmed [31]. This study used the average score across all items, and the Cronbach’s alpha coefficient for the 12-item scale was 0.952.

### 2.4. Data Analysis

The participants’ demographic characteristics and descriptive statistics were calculated for each variable. Next, we compared the Resilience Scale scores, LMX-MDM-J, and distribution of demographic characteristics with and without turnover (binary variable). The Resilience Scale and LMX-MDM-J scores were tested with the independent *t*-test, sex with Fisher’s exact test, and age with the chi-square test. Finally, we conducted binary logistic regression analysis (forced entry method), using the presence or absence of actual turnover as the object variable and the Resilience Scale, LMX-MDM-J, and demographic characteristics (age, sex, and educational background) as explanatory variables. We input only the demographic characteristics in Model 1, demographic characteristics and nurse resilience in Model 2, demographic characteristics and LMX in Model 3, and all variables in Model 4. To see which of the resilience and LMX subscales actually affected actual turnover, in Models 5–8, four subscales of LMX were put in one at a time in addition to demographic characteristics and total resilience scores. Models 9–11 included one of the three resilience subscales in addition to demographic characteristics, and total LMX scores.

ICC and design effects were calculated to examine whether clustering effects needed to be considered. The ICC was 0.016 and the design effect was 1.193. Since a design effect of less than 2 indicates that the effect of clustering is small and does not need to be taken into account [33], this study examined the relationship between actual turnover, resilience, and LMX using logistic regression analysis.

Data analysis for this study was conducted with the consultation of a statistical expert. SPSS Statistics Version 29 (IBM Japan) software was used for the statistical analysis, and the significance level was set at 0.05.

### 2.5. Ethical Considerations

This study was approved by the Institute of Education Ethics Committee of Tokyo Medical and Dental University (Approval No.: C2021-018). Through the nursing department representative of the hospitals that agreed to participate in the survey, questionnaires and explanatory documents were distributed to the participants of Survey 1. The participants were informed in writing about the purpose and methods of this study, the anonymity of their responses, the voluntary nature of their participation, and that there would be no consequences for choosing not to participate. Written informed consent was obtained from all participants.

In this study, it was necessary to assign IDs to individual respondents so that their responses in Survey 1 could be matched with their actual retirement in Survey 2. Therefore, the following measures ensured that researchers could not identify participants. Each participating hospital assigned each subject an ID number and asked them to enter that ID number on the survey form. To ensure that researchers could not identify individuals from the survey forms, each hospital maintained a list of ID numbers and the correspondence between the subject’s name and ID.

## 3. Results

### 3.1. Participant Characteristics

We excluded cases with missing data for the variables used in the binary logistic regression analysis, resulting in a final sample of 1130 individuals (effective response rate: 37.8%). Table 1 presents the participants’ demographic characteristics and descriptive statistics of the variables. The nurses’ mean age was 32.1 years (SD: 9.83), and their average number of years of nursing experience was 10.1 years (SD: 9.11). In terms of education, 49.7% had completed vocational/technical and junior colleges, and 50.3% had graduated from 4-year universities/graduate school. The mean Resilience Scale score was 3.19 (SD: 0.54), and the LMX-MDM-J score was 4.49 (SD: 1.15). Seventy-nine nurses (7.0%) had voluntarily resigned by March 2023.

### 3.2. Bivariate Analysis

Table 2 presents the relationship between actual turnover and nurses’ characteristics, resilience, and LMX. Turnover was significantly associated with years of nursing experience, sex, resilience, and LMX.

### 3.3. Binary Logistic Regression Analysis

Table 3 displays the results of the binary logistic regression analysis using actual turnover as the object variable. In Model 1, which included only the demographic characteristics, nurses in the 25–29 age group were more likely to engage in turnover than those in the 40–63 age group. In Model 2, which incorporated nurse resilience and demographic characteristics, nurses in the 25–29 age group were more likely to engage in turnover than those in the 40–63 age group. In Model 3, which included LMX and demographic characteristics as inputs, LMX significantly influenced actual nurse turnover. In Model 4, which included nurse resilience, LMX, and demographic characteristics, LMX influenced turnover significantly. In Models 5–8, in which demographic characteristics, total resilience scores, and each of the four LMX subscales were fed in, the LMX subscales of loyalty (Model 6), affect (Model 7), and contribution (Model 8) had an effect on turnover. In Models 9–11, in which demographic characteristics, total LMX scores, and each of the three subscales of resilience were input, the three subscales of resilience did not affect turnover.

These results support Hypothesis 2 but not Hypothesis 1. No issues related to multicollinearity were detected among the explanatory variables.

## 4. Discussion

This study investigated whether nurses’ resilience and the quality of the relationship between staff nurses and nurse managers (LMX) influenced turnover. The results showed that nurse resilience was not significantly related to actual turnover; thus, Hypothesis 1 was not supported. The quality of the relationship between staff nurses and nurse managers (LMX) had a significant negative correlation with actual turnover, supporting Hypothesis 2. In other words, LMX has a significant impact on turnover.

### 4.1. LMX and Actual Turnover

In this study, the quality of the relationship between staff nurses and nurse managers (LMX) significantly influenced nurses’ actual turnover. Additionally, the LMX-MDM-J subscales of “loyalty”, “affect”, and “contribution” influenced nurses’ actual turnover. The total LMX score in this study was 4.48 (SD = 1.15), which is similar to the total LMX score of 4.69 (SD = 1.38) in an LMX-MDM-J development study targeting nurses [31]. In a study of nurses conducted in Canada before the COVID-19 pandemic [34], the total LMX score was 4.45 (SD = 1.33). Although this current study was conducted during the COVID-19 pandemic, the scores were similar to those in surveys conducted before the pandemic. In the future, it will be necessary to investigate LMX using the same measurement instrument and to verify and compare it.

Prior research has also shown a relationship between LMX and turnover intentions [35]. However, this study is the first to reveal that LMX is related to reducing actual turnover. The LMX theory considers the relationship of exchanges between staff nurses and nurse managers. To reduce the actual turnover of staff nurses, nurse managers must understand the importance of building high-quality relationships with their subordinates. According to LMX theory, leaders provide psychological rewards to subordinates in the form of trust, respect, and attention [36]. Subordinates, in return, offer trust and loyalty to the leader alongside their work performance. “Loyalty”, a subscale of the LMX-MDM-J, involves being generally consistent and faithful to the individual in a situation and includes items such as “My manager would defend me to others in the organization if I made an honest mistake”, and so on. “Affect” is based on interpersonal attraction rather than work or professional values and includes items such as “My manager is the kind of person one would like to have as a friend”. “Contribution” indicates that the work-related activity fulfills responsibilities beyond the job, completes goals, and includes items such as “I do not mind working my hardest for my manager”. It is important to achieve the LMX subscales of “loyalty”, “affect”, and “contribution”. Specific actions taken by nurse managers using the items that comprise the subscales as cues and guidelines will lead to the curbing of actual turnover.

### 4.2. Nurse Resilience and Actual Turnover

Previous research has shown a relationship between nurses’ resilience and their turnover intention. However, in this study, we could not confirm whether nurses’ resilience influenced actual turnover. The total resilience score in this study was 3.19 (SD = 0.54), which was comparable to 3.35 (SD = 0.52) obtained in the research and development of a Resilience Scale targeting university students [26]. The total Resilience Scale score in a different study targeting nurses [28] was 3.15 (SD = 0.52), approximately the same level as in this study. Generally, resilience is positively correlated with age [37]. Therefore, it was predicted that nurses older than university students would have higher resilience. However, the resilience scores in this study, targeting nurses, were similar to those in a developmental study targeting university students. As this study was conducted during the COVID-19 pandemic, the impact of caring for COVID-19 patients must be considered and examined.

Several mediating variables have been identified in the relationship between resilience and turnover intention. For example, job satisfaction, social support, and job involvement have been found to mediate and influence resilience on turnover intention [38,39,40]. In this study, it is possible that some mediating variable was present, and resilience affected actual turnover through the mediating variable. Future studies should examine the mediating effects of variables associated with turnover in previous studies. Nurse resilience has attributes that include optimism, and the Resilience Scale contains factors that signify expectations of a positive future orientation [26]. Some highly resilient nurses may leave their jobs despite the risks because they hope for the future. Moreover, although it is easy to declare an intention to leave employment, quitting work may be economically and psychologically risky [41].

In a study involving newly graduated nurses, resilience was found to impact actual turnover [42]. In the current study, new graduate and experienced nurses were included, and resilience did not affect actual turnover. Because they had more years of experience than the new graduates, they may have been able to cope with stress even during the COVID-19 pandemic. They likely learned to cope with stress from their experience, and this might not lead them to choose to leave their jobs. There is a need to continue to investigate whether nurse resilience and the three subscales influence actual turnover.

### 4.3. Turnover Rate

We found a turnover rate of 10.2% among nurses at the cooperating hospitals included in this study and a turnover rate of 7.0% among nurses in the analysis. This study’s findings were lower than the overall turnover rate of 11.8% reported by the Japanese Nursing Association [43]. In Japan, national and public hospitals tend to have lower turnover rates than the overall turnover rate [43]. Since two of the advanced treatment hospitals surveyed in this study are national and public institutions, the figures may be lower than the overall turnover rate. It is also possible that the turnover rate is lower because nurses who wanted to leave during the survey did not respond to the questionnaire. Many of the selected advanced treatment hospitals in this study were relatively well resourced, with supplies and knowledge in relation to infection control, even during the COVID-19 pandemic. Therefore, the turnover rates of the three hospitals may have been relatively low compared to other hospitals in Japan.

Nurse turnover rates vary by country but are approximately 10–20% in Korea [44], 20% in Europe and the United States [45,46], and about 30% in the Middle East [47]. In the United States, the turnover rate for other healthcare professionals is reported to be slightly lower than that for nurses, at about 20% [46]. However, in Japan, the turnover rate for nurses in this study is lower than that of the medical and welfare professions, which is 13.3% [48]. Since nurses account for about half of all healthcare professionals [1], organizations need to reduce nurse turnover to maintain healthcare quality.

Although the turnover rate in Japan is lower than in other countries, the fact that a certain number of nurses leave the workforce each year is a situation that cannot be overlooked when addressing the nursing shortage. New nurses must be hired when nurses leave the workforce, and hiring and training them is costly. Staff shortages will occur until the newly hired nurses can care for patients independently without any support from other nurses. When there is a shortage of nurses, the quality of patient care declines. Therefore, organizations and nurse managers must investigate the factors that affect turnover and implement strategies to reduce nurse turnover.

### 4.4. Suggestions for Practice and Theoretical Implications

This study found that although a nurse’s turnover is influenced by age and life events, the quality of the relationship between staff nurses and nurse managers (LMX) led to the suppression of actual turnover. This relationship supports the assertion that nurse manager leadership effectively suppresses staff nurse turnover and that a nurse manager engaged in unit-level management enhances quality relationships with staff nurses. Thus, demonstrating strong leadership by nurse managers may result in the suppression of staff nurse turnover. There is a need to make this outcome known to nurse managers and the heads of the organization to help develop strategies that enable nurse managers to demonstrate leadership. To control nurse turnover, it is also helpful for the nurse manager to take specific actions using the items that comprise the LMX subscale as guidelines for action. The guidelines for action will help the organization and the nurse manager to share action goals for measures to control turnover.

The shortage of nurses is a global problem that threatens patient safety and the quality of medical care that needs to be provided [3]. One way to counter this shortage is to reduce nurse turnover by encouraging nurses to remain in their jobs. This study shows that the quality of the relationship between staff nurses and nurse managers (LMX) influences nurses’ actual turnover and offers measures to policymakers and other decision-makers to prevent nurse turnover.

This study was conducted during the COVID-19 pandemic. Dealing with this unknown infectious disease increased the burden on staff and nurse managers. Research has shown that support and assistance from nursing administrators play an important role in overcoming these situations [49]. The current study showed that nurse managers’ leadership is important in preventing nurses from leaving the profession even in an unstable medical environment such as a pandemic.

In this study, based on previous research on turnover and COR theory, we positioned and tested resilience as a resource and LMX as a relationship that supports the retention and replenishment of resources. The results showed that LMX, a relationship that supports the retaining and replenishing of resources, functions to reduce the actual turnover, thus testing Hypothesis 2. In other words, the LMX may have functioned to retain and replenish resources in COR theory in nurses during the COVID-19 pandemic. Impairment of LMX functionality reduces nurse retention, i.e., loss of LMX functionality may not inhibit nurse turnover. However, the effect of resilience on actual turnover was not found in this study, and there was insufficient evidence for resilience as a resource that inhibits turnover. The impact of resilience on actual turnover should be carefully examined in the future, considering the mediating variables between resilience and actual turnover [38,39,40], as well as attributes such as the experience of individual nurses [42].

During the COVID-19 pandemic, strict infection control was required, face-to-face conferences were discontinued, opportunities for collaboration with other professions were reduced, and restrictions on daily activities reduced opportunities to connect with others and share information. Especially in hospitals during the COVID-19 pandemic, nurses primarily obtained information about the organization from the nurse manager, and the one-on-one relationship between nurses and the nurse manager was emphasized. To ensure that nurses do not choose to leave an organization during a crisis, hospital organizations must support nurse managers to stay connected with nurses and provide leadership.

### 4.5. Limitations and Strengths of This Study and Future Outlook

Our study revealed that nurse managers’ leadership quality influenced their turnover and highlighted the importance of good leadership. This study has three limitations. First, the participants were limited to three advanced treatment hospitals in the Kanto region of Japan. Therefore, this study’s generalizability is limited because it is unknown whether it can be applied to other hospitals. In the future, verifying these findings with nurses who work in other countries, regions in Japan, or hospitals with different functions as study participants will be important. Second, this study was conducted at hospitals with a low nurse turnover rate by nature, that is, advanced treatment hospitals.

Furthermore, nurses with turnover intentions may not have taken the survey during this study. Therefore, the influence of personal and organizational factors related to turnover may be diminished. Further research is needed to determine whether the personal and organizational factors focused on in this study affect nurse turnover in non-advanced treatment hospitals. Finally, since the survey was conducted during the COVID-19 pandemic, the results may have differed from those obtained during less stressful times. Leaders were expected to support nurses psychologically and socially, and nurses may have been psychologically affected.

There are two strengths of this study. First, the explanatory variables, resilience and LMX, and the objective variable, actual turnover, were collected at different times and from different subjects, and the common method bias is considered small. Since the explanatory variables, resilience and LMX, were collected simultaneously and are different concepts, and there was no multicollinearity heteroscedasticity, the relationship between the variables should not affect the results. The second strength of this study is that it is a prospective study that tested the causal relationship between LMX and actual turnover. It is important to continue to investigate the topics of resilience, LMX, and actual turnover among nurses during non-pandemic periods.

## 5. Conclusions

A prospective study of nurses at three advanced treatment hospitals found that LMX (quality of the nurse–nurse manager relationship) impacted actual turnover, whereas resilience had no impact. Specifically, the LMX subscales of “loyalty”, “affect”, and “contribution” had an impact on actual turnover. The LMX needs to be improved to reduce actual turnover among nurses.

## Figures and Tables

**Table 1:** Participants’ characteristics and descriptive statistics (*n* = 1130).

Variables	*n*	Mean or *n*	SD or %
Age (in years)	1130	32.1	9.83
20–24	334		29.56
25–29	269		23.81
30–39	268		23.72
40–63 ^a^	259		22.92
Years of nursing experience	1094	10.1	9.11
NA	36		
Sex	1130		
Female		1021	90.35
Male		109	9.65
Educational background	1130		
Associate degree or below		562	49.73
Bachelor’s degree or higher		568	50.27
Nurse resilience: total score	1130	3.19	0.54
Nurse resilience: novelty seeking		3.17	0.68
Nurse resilience: emotional regulation		3.23	0.58
Nurse resilience: positive future orientation		3.14	0.77
LMX: total score (LMX-MDM-J)	1130	4.49	1.15
LMX: professional respect (LMX-MDM-J)		4.93	1.26
LMX: loyalty (LMX-MDM-J)		4.59	1.33
LMX: affect (LMX-MDM-J)		4.29	1.40
LMX: contribution (LMX-MDM-J)		4.13	1.13
Actual turnover			
Continuing		1051	93.01
Resigned		79	6.99

^a^ Ages 64 and older are not included as they are of mandatory retirement age. LMX: leader–member exchange; LMX-MDM-J: Multidimensional Measure of Leader-Member Exchange, Japanese version.

**Table 2:** Bivariate analysis of turnover (*n* = 1130).

Variables	Continuing (N = 1051)			Resigned (N = 79)			*t*-Value	*p*-Value
	No. of People	Mean	SD or %	No. of People	Mean	SD or %		
Sex								
Female	944		89.82	77		97.47	4.92	0.026 ^b^
Male	107		10.18	2		2.53		
Age (in years)								
20–24	309		29.40	25		31.65		0.00 ^c^
25–29	236		22.45	33		41.77		
30–39	260		24.74	8		10.13		
40–63 ^a^	246		23.41	13		16.46		
Educational background								
Associate degree or below	526		50.05	36		45.57	0.59	0.443 ^c^
Bachelor’s degree or higher	525		49.95	43		54.43		
Years of nursing experience (N = 1095)	1017	10.22	9.12	77	7.92	8.82	−2.14	0.033 ^d^
Nurse resilience: total score	1051	3.20	0.55	79	3.07	0.50	−1.98	0.048 ^d^
LMX: total score (LMX-MDM-J)	1051	4.52	1.15	79	4.10	1.18	−3.12	0.002 ^d^

^a^ Ages 64 and older are not included as they are of mandatory retirement age; ^b^ Fisher’s exact test; ^c^ chi-square test; ^d^ *t*-test. LMX: leader–member exchange; LMX-MDM-J: Multidimensional Measure of Leader-Member Exchange, Japanese version.

**Table 3:** Results of binary logistic regression analysis of turnover (*n* = 1130).

	Model 1			Model 2			Model 3			Model 4		
	Odds Ratio	95% CI		Odds Ratio	95% CI		Odds Ratio	95% CI		Odds Ratio	95% CI	
		LL	UL		LL	UL		LL	UL		LL	UL
Sex (female = 0, male = 1)	0.245	0.059	1.023	0.251	0.060	1.049	0.248	0.059	1.038	0.250	0.060	1.047
Age (in years)												
20–24	1.678	0.795	3.540	1.686	0.798	3.562	1.688	0.796	3.578	1.687	0.796	3.578
25–29	2.965 **	1.418	6.198	2.847 **	1.359	5.961	2.785 **	1.327	5.846	2.732 **	1.300	5.739
30–39	0.660	0.263	1.660	0.656	0.261	1.648	0.602	0.239	1.517	0.604	0.239	1.522
40–63 ^a^	1 (reference)			1 (reference)			1 (reference)			1 (reference)		
Educational background												
Associate degree or below	1.208	0.720	2.026	1.136	0.674	1.917	1.133	0.671	1.912	1.093	0.644	1.855
Bachelor’s degree or higher	1 (reference)			1 (reference)			1 (reference)			1 (reference)		
Nurse resilience: total score				0.688	0.454	1.044				0.804	0.523	1.237
LMX: total score (LMX-MDM-J)							0.736 **	0.608	0.892	0.755 **	0.619	0.922
	**Model 5**			**Model 6**			**Model 7**			**Model 8**		
	**Odds ratio**	**95% CI**		**Odds ratio**	**95% CI**		**Odds ratio**	**95% CI**		**Odds ratio**	**95% CI**	
		**LL**	**UL**		**LL**	**UL**		**LL**	**UL**		**LL**	**UL**
Sex (female = 0, male = 1)	0.251	0.060	1.050	0.244	0.058	1.022	0.248	0.059	1.037	0.259	0.062	1.084
Age (in years)												
20–24	1.777	0.837	3.770	1.713	0.809	3.629	1.596	0.753	3.384	1.558	0.733	3.313
25–29	2.816 **	1.344	5.902	2.810 **	1.340	5.896	2.703 **	1.287	5.675	2.616 **	1.240	5.517
30–39	0.640	0.254	1.610	0.619	0.246	1.557	0.598	0.237	1.508	0.587	0.233	1.482
40–63 ^a^	1 (reference)			1 (reference)			1 (reference)			1 (reference)		
Educational background												
Associate degree or below	1.096	0.647	1.857	1.106	0.653	1.873	1.097	0.647	1.858	1.150	0.678	1.952
Bachelor’s degree or higher	1 (reference)			1 (reference)			1 (reference)			1 (reference)		
Nurse resilience: total score	0.738	0.481	1.132	0.781	0.509	1.200	0.778	0.508	1.191	0.841	0.547	1.292
LMX: professional respect (LMX-MDM-J)	0.870	0.724	1.045									
LMX: loyalty (LMX-MDM-J)				0.814 **	0.684	0.968						
LMX: affect (LMX-MDM-J)							0.781 **	0.663	0.921			
LMX: contribution (LMX-MDM-J)										0.721 **	0.588	0.885
				**Model 9**			**Model 10**			**Model 11**		
				**Odds ratio**	**95% CI**		**Odds ratio**	**95% CI**		**Odds ratio**	**95% CI**	
					**LL**	**UL**		**LL**	**UL**		**LL**	**UL**
Sex (female = 0, male = 1)				0.250	0.06	1.047	0.253	0.060	1.058	0.248	0.059	1.039
Age (in years)												
20–24				1.677	0.791	3.557	1.628	0.766	3.458	1.675	0.787	3.569
25–29				2.708 **	1.288	5.695	2.701 **	1.284	5.682	2.782 **	1.325	5.840
30–39				0.600	0.238	1.512	0.594	0.235	1.497	0.599	0.237	1.513
40–63 ^a^				1 (reference)			1 (reference)			1 (reference)		
Educational background												
Associate degree or below				1.094	0.645	1.855	1.093	0.645	1.852	1.138	0.672	1.926
Bachelor’s degree or higher				1 (reference)			1 (reference)			1 (reference)		
Nurse resilience: novelty seeking				0.839	0.595	1.183						
Nurse resilience: emotional regulation							0.756	0.506	1.129			
Nurse resilience: positive future orientation										1.028	0.752	1.404
LMX: total score (LMX-MDM-J)				0.752 **	0.617	0.916	0.754 **	0.620	0.917	0.733 **	0.601	0.894

** *p* < 0.01; CI: confidence interval, LL: lower limit, UL: upper limit; ^a^ ages 64 and older are not included as they are of mandatory retirement age.

## Data Availability

The datasets mentioned in this article are not currently available as they are part of an ongoing study. Data sharing is not possible for this article since we have not received approval from the participants or Ethics Committee to release the data.
